# Engineered cyanobacteria with additional overexpression of selected Calvin-Benson-Bassham enzymes show further increased ethanol production

**DOI:** 10.1016/j.mec.2021.e00161

**Published:** 2021-01-11

**Authors:** Stamatina Roussou, Alessia Albergati, Feiyan Liang, Peter Lindblad

**Affiliations:** Microbial Chemistry, Department of Chemistry-Ångström, Uppsala University, Box 523, SE-751 20, Uppsala, Sweden

**Keywords:** Cyanobacteria, Calvin-Benson-Bassham, Ethanol, FBP/SBPase, TK, FBA, Carbon fixation

## Abstract

Cyanobacteria are one of the most promising microorganisms to produce biofuels and renewable chemicals due to their oxygenic autotrophic growth properties. However, to rely on photosynthesis, which is one of the main reasons for slow growth, low carbon assimlation rate and low production, is a bottleneck. To address this challenge, optimizing the Calvin-Benson-Bassham (CBB) cycle is one of the strategies since it is the main carbon fixation pathway. In a previous study, we showed that overexpression of either aldolase (FBA), transketolase (TK), or fructose-1,6/sedoheptulose-1,7-bisphosphatase (FBP/SBPase), enzymes responsible for RuBP regeneration and vital for controlling the CBB carbon flux, led to higher production rates and titers in ethanol producing strains of *Synechocystis* PCC 6803. In the present study, we investigated the combined effects of the above enzymes on ethanol production in *Synechocystis* PCC 6803.

The ethanol production of the strains overexpressing two CBB enzymes (FBA ​+ ​TK, FBP/SBPase ​+ ​FBA or FBP/SBPase ​+ ​TK) was higher than the respective control strains, overexpressing either FBA or TK. The co-overexpression of FBA and TK led to more than 9 times higher ethanol production compared to the overexpression of FBA. Compared to TK the respective increase is 4 times more ethanol production. Overexpression of FBP/SBPase in combination with FBA showed 2.5 times higher ethanol production compared to FBA. Finally, co-overexpression of FBP/SBPase and TK reached about twice the production of ethanol compared to overexpression of only TK. This study clearly demonstrates that overexpression of two selected CBB enzymes leads to significantly increased ethanol production compared to overexpression of a single CBB enzyme.

## Highlights

• ^**.**^ Effects of CBB enzymes expression on ethanol yield in *Synechocystis* PCC 6803 analyzed.
• ^**.**^ Increased ethanol production when overexpression two selected CBB enzymes.
• ^**.**^ Highest ethanol production in strain overexpressing a combination of FBA and TK.

## 1 Introduction

Since the industrial revolution, coal and other fossil resources have been major energy sources (Newman et al., 2017). This has caused serious environmental issues, including the release and high level of CO_2_ in the atmosphere as well as pollution (Deneyer et al., 2018). Therefore, more environmentally friendly renewable energy sources, such as biofuels are urgently needed to be developed (Zhou et al., 2018). This includes products that derive from biomass or its residuals, as it can be bio-ethanol (Lin et al., 2014). Currently, there are four generations of biofuels. The first two generations include heterotrophic microorganisms using agricultural byproducts as energy source; one example is yeast, while the last two are based on photosynthetic microorganisms as photoautotrophic algae and cyanobacteria (Callegari et al., 2020). Compared to the first two generations, they require mainly solar energy and CO_2_ for growth. Therefore they can be cultivated on free arable land avoiding competition with e.g. the food industry (Zahra et al., 2020) which makes them promising green cell factories (Repice et al., 2010).

Cyanobacteria are one of the oldest microorganisms on earth (Kasting and Siefert, 2002) and furthermore, they are vital for the oxygen cycle (Nadis, 2003). Due to the research progress in synthetic and molecular biology, gene modification technologies become mature and ready (Jin et al., 2019; Heidorn et al., 2011). The model organism *Synechocystis* PCC 6803, a unicellular cyanobacterium, has been engineered to produce numerous chemicals and fuels from solar energy and CO_2_ (Deviram et al., 2020). Despite the development, there are still obstacles to overcome such as low growth rate and low production (Yu et al., 2013). Furthermore, CO_2_ fixation and further channeling the fixed carbon into selected products are among the identified bottlenecks (Luan et al., 2020; Liu et al., 2019).

**Fig. 1:**
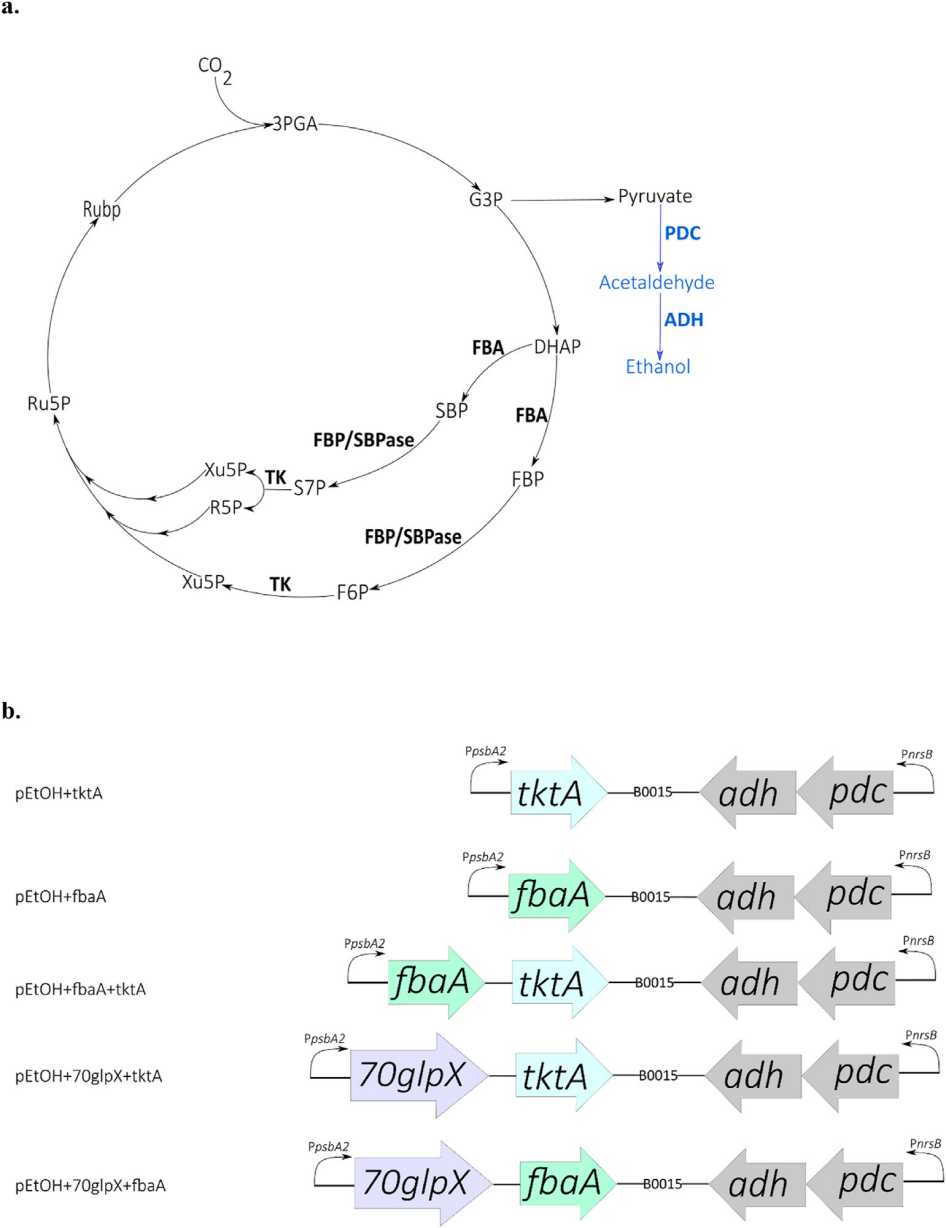
Simplified outline of the Calvin-Benson-Basshmam (CBB) cycle with selected key enzymes indicated (**a**) and corresponding genetic constructs used in the present study (**b**). The CBB cycle is represented by black color and black lines while the inserted ethanol pathway by blue color and blue lines (**a**). The overexpressed enzymes are written in bold form and intermediates using abbreviations; 3PGA, 3-phosphoglycerate; G3P, glyceraldehyde-3-phosphate; DHAP, dihydroxyacetone phosphate; FBP, fructose-1,6-bisphosphate; SBP, sedoheptulose-1,7-bisphosphate; F6P, fructose-6-phosphate; S7P, sedoheptulose-7-phosphate; Xu5P, xylulose-5-phosphate; R5P, ribose-5-phosphate; Ru5P, ribulose-5-phosphate; RuBP, ribulose-1,5-bisphosphate. Enzymes; FBA, aldolase; FBP/SBPase, fructose-1,6/sedoheptulose-1,7-bisphosphatase; TK, transketolase; PDC, pyruvate carboxylase; ADH, alcohol dehydroge. The expression constructs were inserted in the plasmid vector pPMQAK1 (**b**). P*nrsB* and P*psbA2* represent the promoters and B0015 the terminator used. The ethanol pathway includes the genes pyruvate carboxylase (*pdc*) originated from *Zymomonas mobilis* and alcohol dehydrogenase (*adh*) from *Synechocystis* PCC 6803. The selected, overexpressed CBB genes were transketolase (*tktA*), aldosase (*fbaA*), and FBP/SBPase (*70glpX*). *tktA* and *fbaA* are the native genes of *Synechocystis* PCC 6803 while *70glpX* is the homologue from the strain *Synechococcus* PCC 7002. (For interpretation of the references to color in this figure legend, the reader is referred to the Web version of this article.)

The main carbon fixation pathway in cyanobacteria is the Calvin Benson-Bassham (CBB) cycle, which follows the light reaction of photosynthesis (Fig. 1a). It contains thirteen reactions organized in three parts: 1. Ribulose-1, 5-bisphosphate (RuBP) carboxylation, 2. 3-phosphoglycerate (PGA) reduction and 3. RuBP regeneration, which is the part that includes most of the chemical reactions (Liang et al., 2018b). The cycle includes eleven enzymes and studies have identified four of them which show control over the carbon flux: ribulose-1, 5-bisphosphate carboxylase/oxygenase (RuBisCO), aldolase (FBA), the bifunctional enzyme fructose-1,6-/seduheptoluse-1,7-biphosphatase (FBP/SBPase) and transketolase (TK) (Zhu et al., 2007). The last three of them are enzymes for the regeneration of RuBP.

In several studies, selected CBB enzymes have been overexpressed in either plants or cyanobacteria. Overexpression of the native aldolase led to enhanced activity of the enzyme and increased fixation of CO_2_ when tested in *Arabidopsis thaliana* (Hatano-Iwasaki and Ogawa, 2012; Simkin et al., 2017). Overexpression of the aldolase originated from *Synechocystis* PCC 6803 in both the green algae *Chlorella vulgeris* and the origin strain, led to increased growth rates and biomass accumulations (Yang et al., 2017; Liang and Lindblad, 2016).

The bifunctional FBP/SBPase of cyanobacteria has also been overexpressed in plants and cyanobacteria. The results in plants showed increased carbon fixation and growth (Miyagawa et al., 2001; Simkin et al., 2017). A similar pattern was observed when the enzyme was overexpressed in cyanobacteria (Liang and Lindblad, 2016; De Porcellinis et al., 2018). Less is known about transketolase (TK). A study on overexpression of TK in tobacco plants showed higher level of the enzyme’s activity but lower level of thiamine (Khozaei et al., 2015) while overexpression of the enzyme in *Synechocystis* PCC 6803 resulted in increased growth and biomass formation (Liang and Lindblad, 2016).

Cyanobacteria have been used for the production of bio-ethanol (Deng and Coleman, 1999; Dexter and Fu, 2009). Ethanol is among the most promising candidates for biofuel production as it shows a higher octane number and higher evaporation rate compared to gasoline (Balat, 2007). Furthermore, the process to engineer cells to produce ethanol is simple, only two additional enzymes are required for the production; pyruvate decarboxylase (PDC) and alcohol dehydrogenase (ADH). In a previous study, we demonstrated that overexpression of one of the selected four enzymes of the CBB cycle in combination with expression of the ethanol pathway, led to increased production in the engineered *Synechocystis* PCC 6803 strains (Liang et al., 2018a). The best producing strain was the strain overexpressing FBA, followed by the strains overexpressing FBP/SBPase and RubisCO.

In the current study, we investigated the effects of overexpressing two CBB enzymes in an ethanol producing strain of *Synechocystis* PCC 6803. We focused on the enzymes FBA, TK and FBP/SBPase in order to examine if the productivity can be enhanced when the carbon flux on the regeneration of RuBP is enhanced by a combined overexpression of two of these enzymes. The higher increased in ethanol production was observed in the strain overexpressing FBA and TK. Increased ethanol production was also noticed in the strain overexpressing FBP/SBPase and TK.

## 2 Materials and methods

### 2.1 Strains and cultivation conditions

LB medium was used to grow *Escherichia coli* (*E. coli*) strains, and the cells were cultivated in liquid or 1.5% agar (w/v) plates under 37 ​°C. *Synechocystis* PCC 6803 (*Synechocystis*) was cultivated in BG11 (Stanier et al., 1971), under 30 ​°C and 50 ​μmol photons m^−2^ s^−1^ in liquid or 1.5% agar (w/v) plates, both the wild type and the engineered strains. The selective antibiotic was kanamycin with a final concentration 50 ​μg/ml, for both *E. coli* and *Synechocystis*.

### 2.2 Plasmid construction and conjugation of *Synechocystis*

The plasmids used in this study are shown in Table 1 and Fig. 1b. The genetic constructs overexpressing two selective Calvin-Benson-Bassham Cycles (CBB) genes were based on previously described plasmids (Liang et al., 2018a). In details, the plasmids pEtOH-tktA, pEtOH-fbaA were used as templates to amplify the genetic regions rbs (ribosomal binding site)-tktA-B0015 (terminator) and rbs-fbaA-B0015 with corresponding primers (Table 2) and the polymerase Phusion Polymerase (Thermo Fisher Scientific). In order to amplify P*psbA2*-fbaA and P*psbA2*-70glpX, with corresponding primers, the plasmids pEtOH-fbaA and pEtOH-70glpX were used as templates, respectively. The PCR products were digested with the necessary restriction enzymes (*EcoRI*, *PstI*, *XbaI* and *BcuI*), ligated into the plasmid pEtOH and transformed to competent *E. coli* cells. All restriction enzymes were from Thermo Fisher Scientific.

**Table 1:** Characteristics of engineered *Synechocystis* PCC 6803 strains used (*Synechocystis* strains).

Plasmids	Promoters and enzymes	*Synechocystis* strains	References
pEtOH ​+ ​fbaA	P*nrsB*, pyruvate decarboxylase (PDC) and alcohol dehydrogenase (ADH); P*psbA2*, aldolase (FBA)	EtOH ​+ ​fbaA	Liang et al. (2018a)
pEtOH ​+ ​tktA	P*nrsB*, PDC and ADH; P*psbA2*, transketolase (TK)	EtOH ​+ ​tktA	Liang et al. (2018a)
pEtOH ​+ ​fbaA ​+ ​tktA	P*nrsB*, PDC and ADH; P*psbA2*, FBA and TK	EtOH ​+ ​fbaA ​+ ​tktA	This study
pEtOH+70glpX ​+ ​tktA	P*nrsB*, PDC and ADH; P*psbA2*, fructose-1,6-/sedoheptulose-1,7-bisphosphatase (FBP/SBPase) and TK	EtOH+70glpX ​+ ​fbaA	This study
pEtOH+70glpX ​+ ​fbaA	P*nrsB*, PDC and ADH; P*psbA2*, FBP/SBPAse and FBA	EtOH+70glpX ​+ ​tktA	This study

**Table 2:** Primers used in this study.

Primer	Sequence
**tktA_Xb∗_F**	GATATCTAGATAGTGGAGGTT … TGGTCGTTGCTACCCAGTC
**tktA_seq_F1**	CTTTCGGCCCTTTCCGAG
**tktA_sta_R**	CAAAGCGGTCCCGATTGAAC
**70glpx_SP_R**	CTCTGCAGCTATACTAGTCTAGAGTTGAATATTTTGGGGG
**70glpX_seq_F**	GGCAGTCCTCGCCATTTC
**70glpX_sta_R**	GAAATGGCGAGGACTGCC
**70glpX_seq_F1**	GTTCCATGGTGGCGCAC
**70glpx_Xb∗_F**	GATATCTAGATAGTGGAGGTTACTAGGTGGAAAGCACCCTCGG
**fbaA_Xb∗_F**	GATATCTAGATAGTGGAGGTTACTAGATGGCTCTTGTACCAATGAGAC
**fbaA_end_R**	CAATTGACCTTGGATGACTAC
**fbaA_sta_R**	CAAGGAACCGTCCATCATC
**TB15_R**	GACTGCAGTATAAACGCAGAAAGGCCCAC
**PpsbA2_Eco_F**	GAGAGAATTCCCGCCAGGTAAACTCTTCTC
**SR-slr1192OP-seq-F**	CACCATGTTAGATTTTGCCG
**pPMQAK1_SF**	CTCGTGCACCCAACTGATCTTCAGCATC

The *E. coli* cargo cells and *E. coli* helper cells (*E. coli* HB101) carrying the plasmid RL443-AmpR were cultivated overnight, centrifuged at 3000 ​rpm for 5 ​min and resuspended in 1 ​ml fresh LB medium without antibiotics. The wild type *Synechocystis* strain (200 ​μl) was mixed with 1 ​ml cargo cells and 1 ​ml helper cells. The mixture was incubated under 100 ​μmol photons m^−2^ s^−1^ for 1.5 ​h and then was spread on a filter (GN-6 Metricel, 0.45 ​μm, 47 ​mm) on a BG11 plate without antibiotics for 48 ​h incubation. Then the filter was transferred on a BG11 plate with the corresponding antibiotic and the candidate colonies were analyzed with colony PCR.

### 2.3 Ethanol production and quantification

In order to grow the engineered *Synechocystis* cells for ethanol production, two different growth systems were used. First, the cells needed for the inoculation of the production experiment, were cultivated in 6-well Tissue Culture plates (Sarsedt) in a total volume of 4 ​ml BG11 until the mid-log phase. For the production experiment, the cells were cultivated in 20 ​ml BG11, 50 ​mM NaHCO_3_ under 50 ​μmol photons m^−2^ s^−1^ in closed system with the use of Biolite flasks (Plug Seal, Thermo Scientific) to prevent any ethanol evaporation. The starting optical density (OD_730_) was 0.1 and the cells were grown in three biological replicates for 20 days. Every second day the pH was adjusted (7.5) with the addition of 37% HCl (Sigma-Aldrich) and NaHCO_3_ was added (50 ​mM final concentration). These two steps were vital for the successful cultivation of the cells as when HCO_3^−^_ is diffused in the carboxysome, it leads to the increase of CO_2_ close to RubisCO (Durall et al., 2020) and increased concentration of OH^−^ and eventually alkalization of the pH, that leads to the starvation of the cells. To induce the P*nrsB* promoter, and thereby the ethanol production, 2.5 ​μM of Ni^2+^ (NiCl_2_) was used and it was added in the cultures every second day.

To quantify the ethanol production, a 1 ​ml sample was pelleted and 1 ​μl of the supernatant was analyzed with a Clarus 580 Perkin Elmer FID gas chromatograph (GC) which has a packed column (1.8 ​m ​× ​2 ​mm i.d., Cat No. N9305013-ZW5531, Perkin Elmer). The carrier gas was N_2_, 20 ​ml ​min^−1^. The GC program used was 130 ​°C for 5.5 ​min, followed by heating up to 230 ​°C (the rate of increase was 45 ​°C min^−1^) for 5 ​min. The ethanol peak appeared at 4.96 ​min. External standard curve was used to quantify ethanol production (R^2^ ​> ​0.99). To measure the optical density, 100 ​μl from the culture were sampled.

## 3 Results

### 3.1 Over-expressing TK or FBP/SBPase in combination with FBA leads to increased level of ethanol production

**Fig. 2:**
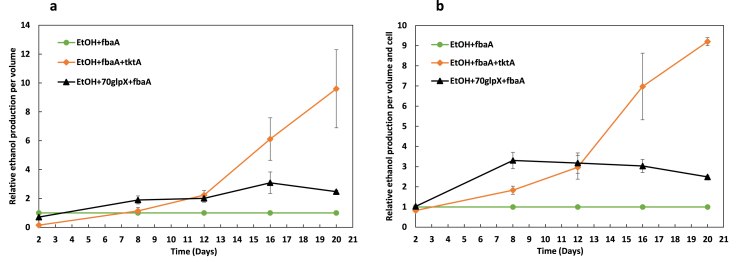
Ethanol production in *Synechocystis* PCC 6803 strains engineered to contain the ethanol pathway (EtOH; PDC and ADH), and overexpressing one (FBA) or two (FBA ​+ ​TK, FBP/SBPase ​+ ​FBA) enzymes of the CBB cycle. Shown are the relative ethanol production per volume (**a**) and per volume and cell (**b**) for the strains EtOH ​+ ​fbaA ​+ ​tktA and EtOH+70glpX ​+ ​fbaA compared to the control strain (EtOH ​+ ​fbaA). The strains were cultivated under 50 ​μmol photons m^−2^ s^−1^ while every two days NaHCO_3_ with final concentration 50 ​mM was added to the culture as well as 2.5 ​μM Ni^+2^ to induce the ethanol production. In the same day the pH was adjusted with HCl (7.5). Ethanol content and OD were measured on days 2, 8, 12, 16 and 20. The results are represented as mean ​± ​SD of the three biological replicates.

In a previous study, we demonstrated higher production of ethanol when FBA was overexpressed (Liang et al., 2018a). In the present study, FBA was overexpressed in combination with a selected second enzyme of the CBB cycle, TK or FBP/SBPase. The plasmid pPMQAK1 was engineered to contain the ethanol pathway as an operon driven by the inducible promoter P*nrsB* and the CBB genes were added in the opposite direction as an operon under the native promoter P*psbA2*. For the strain EtOH ​+ ​fbaA ​+ ​tktA, the FBA gene was directly following the promoter while the gene coding for FBP/SBPase enzyme was directly under the promoter for the strain EtOH+70glpX ​+ ​fbaA (Fig. 1b). At the end of the cultivation period, significantly higher production of ethanol was observed (Fig. 2). In order to visualize the effect of overexpressing a second CBB enzyme, the results are presented as the relative production of ethanol either per volume or per volume and cell. The production of the control stain with an overexpression of FBA only (EtOH ​+ ​fbaA) was normalized as 1.

The strain overexpressing both FBA and TK (EtOH ​+ ​fbaA ​+ ​tktA) showed 9.6 times more ethanol production per volume (Fig. 2a), with an ethanol titer of 1.2 ​g/L on the last day of the experiment. Increased production was also noticed from the strain overexpressing FBP/SBPase and FBA (EtOH+70glpX ​+ ​fbaA), which was up to 2.5 times higher compared to the EtOH ​+ ​fbaA strain (Fig. 2a). It is noticed that both strains overexpressing two CBB enzymes showed less ethanol production compared to the control strain on day 2 but afterwards, on days 8, 12, 16 and 20 both of them produced more ethanol. The relative ethanol production normalized per cell (Fig. 2b) followed similar patterns, 9.2 times increase for the strain EtOH ​+ ​fbaA ​+ ​tktA and 2.5 for the strain EtOH+70glpX ​+ ​fbaA, both of them compared to the control strain EtOH ​+ ​fbaA.

### 3.2 Over-expressing FBA or FBP/SBPase in combination with TK leads to higher level of ethanol production

**Fig. 3:**
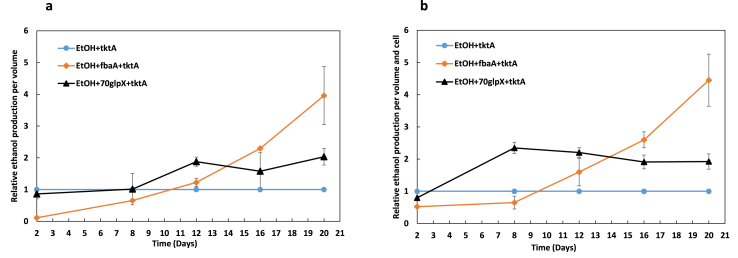
Ethanol production in *Synechocystis* PCC 6803 strains engineered to contain the ethanol pathway (EtOH; PDC and ADH) and overexpressing one (FBA) or two (FBA ​+ ​TK, FBP/SBPase ​+ ​TK) enzymes of the CBB cycle.Shown are the relative ethanol production per volume (**a**) and per volume and cell (**b**) for the strains EtOH ​+ ​fbaA ​+ ​tktA and EtOH+70glpX ​+ ​tktA compared to the control strain (EtOH ​+ ​fbaA). The strains were cultivated under 50 ​μmol photons m^−2^ s^−1^ while every two days NaHCO_3_ with final concentration 50 ​mM was added to the culture as well as 2.5 ​μM Ni^+2^ to induce the ethanol production. In the same day the pH was adjusted with HCl (7.5). Ethanol content and OD were measured on days 2, 8, 12, 16 and 20. The results are represented as mean ​± ​SD of the three biological replicates.

It is known from earlier studies (Liang et al., 2018a) that higher level of ethanol production is noticed when TK is overexpressed. In this study, we observed that overexpressing TK in combination with FBA or FBP/SBPase led to even higher ethanol production. For the strain EtOH+70glpX ​+ ​tktA, the FBP/SBPase coding gene was under the direct control of the promoter and the transketolase gene was the second gene in the operon (Fig. 1b). During this study two strains overexpressing tktA and one more CBB gene (EtOH ​+ ​fbaA ​+ ​tktA and EtOH+70glpX ​+ ​tktA) were compared to the control strain EtOH ​+ ​tktA. The strain EtOH ​+ ​fbaA ​+ ​tktA produced approximately 4 times more ethanol compared to EtOH ​+ ​tktA at the end of the cultivation period (Fig. 3). The strain EtOH+70glpX ​+ ​tktA produced the double amount of ethanol compared to EtOH ​+ ​tktA (Fig. 3).

It is noticeable that on day 2 the relative ethanol production of the examined strains were lower than in the control strain (EtOH ​+ ​tktA).

### 3.3 The combined overexpression of FBA and TK leads to similar production patterns against overexpression of FBA or TK individually

**Fig. 4:**
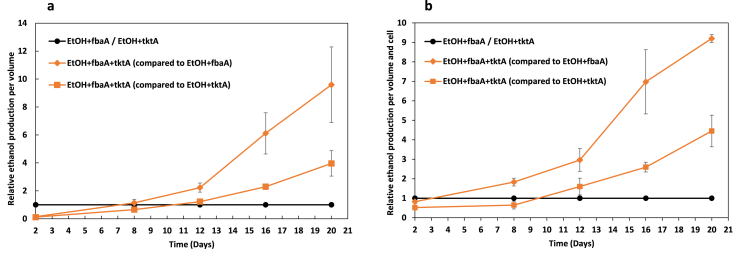
Ethanol production in *Synechocystis* PCC 6803 strains engineered to contain the ethanol pathway (EtOH; PDC and ADH) and overexpressing two (FBA ​+ ​TK) enzymes of the CBB cycle. Shown are the relative ethanol production per volume (**a**) and per volume and cell (**b**) for the strain EtOH ​+ ​fbaA ​+ ​tktA compared to the control strain EtOH ​+ ​fbaA or the control strain EtOH ​+ ​tktA. The strains were cultivated under 50 ​μmol photons m^−2^ s^−1^ while every two days NaHCO_3_ with final concentration 50 ​mM was added to the culture as well as 2.5 ​μM Ni^+2^ to induce the ethanol production. In the same day the pH was adjusted with HCl (7.5). Ethanol content and OD were measured on days 2, 8, 12, 16 and 20. The results are represented as mean ​± ​SD of the three biological replicates.

Overexpression of FBA and TK individually showed increased ethanol production (Liang et al., 2018a) while the combined overexpression of the two enzymes reached even higher ethanol yield. The best producing strain was EtOH ​+ ​fbaA ​+ ​tktA, both in absolute ethanol titer production and in relative ethanol production compared to either EtOH ​+ ​fbaA or EtOH ​+ ​tktA. We noticed that the strain showed similar behavior compared to each respective control strain (Fig. 4). On day 2, the EtOH+fbaA+tktA produced less ethanol compared to the control strains while on day 8 ethanol production was similar and after day 12, the difference in the production level was clear.

## 4 Discussion

Increasing the carbon fixation is one of the promising solutions to address the low production yield occurring not only in cyanobacteria but also in plants. Studies show that overexpressing selected Calvin-Benson-Bassham (CBB) enzymes increases the production rate in both organisms (Liang et al., 2018b; Ogawa et al., 2015). The overexpression of FBP/SBPase originated from *Synechococcus elongatus* PCC 7942 in the algal *Euglena gracilus*, led to increased level of ester production under specific conditions (Ogawa et al., 2015). Furthermore, overexpression of selected CBB enzymes in *Synechocystis* led to increased level of both product (ethanol) and biomass indicating that the overall performance of the carbon fixation pathway was significantly increased (Liang et al., 2018a). In addition, strains containing the ethanol pathway and overexpressing a single CBB gene showed reduced growth compared to the strain only expressing the ethanol pathway. The same pattern was observed in the present study, e.g. strain EtOH ​+ ​fbaA ​+ ​tktA showed 25% lower growth compared to the strain EtOH ​+ ​fbaA, and 30% reduction compared to EtOH ​+ ​tktA. Additionally, strain EtOH+70glpX ​+ ​fbaA showed 20% lower growth compared to strain EtOH ​+ ​fbaA, and strain EtOH+70glpX ​+ ​tktA showed 12% reduced growth compared to strain EtOH ​+ ​tktA.

Overexpression of FBP/SBPase and FBA led to increased level of ethanol production of 67% and 69% more respectively, while overexpression of TK led to 37% more ethanol compared to the strain expressing only the ethanol pathway (Liang et al., 2018a). These three enzymes regulate the carbon flux (Zhu et al., 2007) and are part of the regeneration of RuBP in the CBB cycle (Liang et al., 2018a, Liang et al., 2018b). In addition, these enzymes are participating in more metabolic reactions of the cell. FBA is involved in the oxidative pentose phosphate while FBP/SBPase is involved in glyconeogenesis (Liang et al., 2018a, Liang et al., 2018b). TK is important for both primary and secondary metabolism and it is found in oxidative pentose pathway (OPP) (Bi et al., 2013).

In the present study, we examined the effect of the combined overexpression of selected enzymes of the CBB enzymes in the formation of ethanol and we optimized the growth conditions of *Synechocystis*. In addition, every second day the pH was adjusted to 7.5 with the use of 37% HCl as previously result has shown that this is the most efficient way to adjust the pH in the culture (Miao et al., 2018).

Our data show that overexpressing two CBB enzymes reach even higher level of ethanol production. The co-overexpression of FBA and TK reached approximately 9 times more ethanol production compared to the strain overexpressing FBA and 4 times more compared to the one overexpressing TK (Fig. 4). Metabolomic analysis has shown that overexpression of TK led to decrease of dihydroorotate oxidase, a key enzyme for the biosynthesis of pyrimidine rings (Hing et al., 2019). The same analysis also demonstrated that overexpression of the enzyme may cause oxidative stress and pigment degradation. Furthermore, the strain EtOH ​+ ​fbaA showed slightly increased doubling time compared to the ethanol-producer strain (Douchi et al., 2019). These observations may explain that until day 12, the strain produced the same or slightly more ethanol compared to the control strains (Fig. 4). However, on the last day of the experiment, the production titer was 1.2 ​g/L, corresponding to 28 ​mM ethanol, and the relative production was much higher compared to the control strains. Interestingly, in the original report on ethanol producing cells of *Synechocystis* PCC 6803, the ethanol concentration peaked at round 12 ​mM and the cells could tolerate up to 1% (about 230 ​mM) exogenously added ethanol (Dexter and Fu, 2009). MIMS analysis demonstrated that the EtOH ​+ ​fbaA strain showed that overexpression of FBA can equilibrate the loss of carbon that is used for ethanol production. This fact may also explain the increased ethanol production in the last days of the experiment (Douchi et al., 2019). Finally, both TK and FBA are involved in the OPP pathway so when both of them are overexpressed, the pathway does not seem to show the reduction of activity which has been observed when only the TK is being overexpressed (Hing et al., 2019). The combined overexpression of TK and FBA may equilibrate the reduction in the OPP pathway but other, or additional, mechanisms cannot be excluded. However, since TPP/Thiamine is a co-factor for both the CBB enzymes and in the ethanol step from pyruvate, the availability of TPP/Thiamine may become a limiting factor when engineering cyanobacteria for increased ethanol synthesis.

The combined overexpression of FBA and FBP/SBPase led to 2.5 times higher production of ethanol compared to in the control strain EtOH ​+ ​fbaA (Fig. 2). In agreement, the overexpression of FBA and SBPase in tobacco plants resulted in increased photosynthesis and rate of CO_2_ assimilation (Simkin et al., 2015). The ethanol production per volume is lower on day 2 compared to in the control stain while the ethanol production per volume and cell is almost the same. This may be explained by the small increased doubling time noticed for the strain EtOH ​+ ​fbaA (7.1/h compared to 6.4/h for the WT strain) and the reduced levels of the OPP enzymes (Douchi et al., 2019; Hing et al., 2019). Later in the cultivation period, the production is higher compared to the control strain so the increased levels of two CBB enzymes result in higher efficiency of the CBB cycle.

The additional expression of FBP/SBPase enzyme led to approximately 2 times higher level of ethanol production compared to in the control strain EtOH ​+ ​tktA (Fig. 3). Again, we noticed that on day 2 the ethanol production was lower than in the control strain, indicating that the overexpression of two enzymes causes a temporary stress condition in the cells. This may be overcome by overexpressing carbon transporters with the aim to enhance the influx of carbon into the cells. Cyanobacteria have five different inorganic carbon (Ci) transporters (Liang et al., 2018b). Only one of them, BicA, has been overexpressed in *Synechocystis*, and corresponding strain showed enhanced carbon fixation when a higher carbon concentration was provided to the cells (discussed in Liang et al., 2018b). Overexpressing higher CO_2_ affinity transporters may increase growth under ambient CO_2_ conditions. An additional strategy may be to change low Ci induced transporters, like SbtA, BCT1 and NDH-I3, into constitutively expressed transporters. However, as the cultivation of the cells in the present study continued up to 20 days we observed increased yield of production clearly demonstrating that the overexpression of two selected CBB enzymes enhances the ethanol formation. Additional genetic engineering may further increase this production.

## CRediT authorship contribution statement

**Stamatina Roussou:** Methodology, Investigation, Writing - original draft, Writing - review & editing. **Alessia Albergati:** Investigation, Writing - review & editing. **Feiyan Liang:** Conceptualization, Methodology, Writing - review & editing, Visualization. **Peter Lindblad:** Conceptualization, Writing - review & editing, Supervision, Funding acquisition.

## Declaration of competing interest

There are no conflicts to declare.
